# Store-operated calcium entry and the localization of STIM1 and Orai1 proteins in isolated mouse sinoatrial node cells

**DOI:** 10.3389/fphys.2015.00069

**Published:** 2015-03-09

**Authors:** Jie Liu, Li Xin, Victoria L. Benson, David G. Allen, Yue-Kun Ju

**Affiliations:** ^1^School of Medical Sciences and Bosch Institute, University of SydneySydney, NSW, Australia; ^2^Victor Chang Cardiac Research InstituteSydney, NSW, Australia

**Keywords:** store-operated Ca^2+^ entry, STIM1, Orai1, sinoatrial node

## Abstract

In many non-excitable and excitable cells, store-operated calcium entry (SOCE) represents an additional pathway for calcium entry upon Ca^2+^ store depletion. In a previous study, we demonstrated SOCE activity in intact mouse cardiac pacemaker tissue, specifically from sinoatrial node (SAN) tissue. However, store content as a key determinant of SOCE activity is difficult to measure in intact SAN tissue. Therefore, to investigate the interaction between SOCE and store content and its role in cardiac pacemaking, it is necessary to investigate SOCE activity in single cardiac pacemaker cells. Furthermore, recent studies in other tissues have identified two new proteins involved in SOCE, stromal interacting molecule (STIM), which is an ER Ca^2+^ sensor, and the surface membrane channel Orai, a prototypic gene encoding for SOCE. However, whether STIM and Orai are expressed in native pacemaker cells is still unknown. In this current study, we examined SOCE activity in single firing pacemaker cells isolated from mouse sinoatrial node tissue. We found a significant rise in Ca^2+^ entry in response to Ca^2+^ store depletion. SOCE blockers reduced the amplitude and frequency of spontaneous Ca^2+^ transients and reduced Ca^2+^ store content. We demonstrated for the first time that STIM and Orai are expressed in pacemaker cells. After store depletion, STIM1 redistributed to the cell periphery and showed increased co-localization with surface membrane located Orai1, indicating a possible involvement of these proteins in SOCE activity in native cardiac pacemaker cells. These results suggest the novel concept that SOCE plays a functional role in regulating intracellular Ca^2+^ of cardiac pacemaker cells.

## Introduction

Cardiac contraction originates in the spontaneous firing of pacemaker cells in the sinoatrial node (SAN) of the heart. Originally it was thought that the spontaneous firing of pacemaker cells was driven purely by voltage-dependent membrane currents (Noble, 1960) but subsequently it has been found that intracellular Ca^2+^ cycling also plays an important role (Rigg and Terrar, 1996; Ju and Allen, 1998, 1999; Rigg et al., 2000; Lakatta et al., 2010). During each spontaneous cycle, Ca^2+^ influx through L-type Ca^2+^ channels triggers sarcoplasmic reticulum (SR) Ca^2+^ release and produces a global Ca^2+^ transient. The loss of Ca^2+^ from the SR causes partial depletion which recovers as the SR Ca^2+^ pump (SERCA) returns Ca^2+^ to the SR. Ca^2+^ extrusion through the Na^+^/Ca^2+^ exchanger generates an inward current that contributes to pacemaker diastolic depolarization. (Rigg and Terrar, 1996; Ju and Allen, 1998, 2000; Rigg et al., 2000; Vinogradova et al., 2000, 2006). Although controversies still exist about the relative importance of intracellular Ca^2+^ cycling over membrane currents (Lakatta and DiFrancesco, 2009; Himeno et al., 2011), it is now generally accepted that pacemaker activity is orchestrated by the coupled system of membrane ionic currents (the “membrane clock”) and intracellular SR calcium cycling (the “calcium clock”) (for review see, Lakatta et al., 2010).

In many non-excitable and excitable cells, store-operated calcium entry (SOCE) represents an additional pathway for calcium entry into the cell to refill the SR calcium store. SOCE is activated by the decline of Ca^2+^ concentration within the lumen of the endoplasmic reticulum (ER) or SR. Activation of SOCE produces Ca^2+^ influx which provides a regulatory link between SR Ca^2+^ level and membrane calcium entry, thereby helping to maintain SR calcium homeostasis (Parekh and Putney, 2005). In a previous study, we demonstrated SOCE in an intact SAN tissue preparation based on a rise of intracellular Ca^2+^ ([Ca^2+^]_*i*_) when extracellular Ca^2+^ was replenished after store depletion (Ju et al., 2007). However, SAN tissue contains pacemaker cells intermingled with fibroblasts, atrial myocytes and endothelium cells, all of which are capable of expressing SOCE (Camelliti et al., 2004; Chen et al., 2010; Li et al., 2011). Additionally, store content as a key determinant of SOCE activity is difficult to measure in intact SAN tissue. Therefore, to rule out possible endogenous cell contamination and investigate store content, it is necessary to investigate SOCE activity in single pacemaker cells.

In the present study, we examined SOCE in single firing pacemaker cells, by measuring the changes in intracellular Ca^2+^ in response to SR Ca^2+^ store depletion. Using two different blockers of SOCE, SKF96365, and BTP-2, we estimated the contribution of SOCE to pacemaker activity and the refilling of the SR Ca^2+^ store.

The genes encoding SOCE in pacemaker cells remain to be identified. We previously found that TRPC channel genes are expressed in pacemaker cells isolated from mouse SAN. Because the involvement of TRPC channels in SOCE has also been reported in many other cell types (Clapham, 2003; Birnbaumer, 2009), this result suggests that TRPC channels could be encoding SOCE in pacemaker cells. Ca^2+^ influx through TRPC channels has also been implicated in cardiac hypertrophy (Wu et al., 2010; Eder and Molkentin, 2011) and cardiac arrhythmias (Harada et al., 2012). However, despite the large numbers of reports implicating TRPCs as store-operated channels, whether activation of TRPC channels requires depletion of SR Ca^2+^ store remain debatable (Lewis, 2007; Birnbaumer, 2009; Putney, 2009). Accumulated evidence suggests that activation of certain isoforms of TRPC channels, especially TRPC3 and TRPC6, are directly related to G-protein coupled receptor activation and phospholipase C mediated production of diacylglycerol, referred to as receptor-operated Ca^2+^ entry (ROCE) (Hofmann et al., 1999; Onohara et al., 2006; Mohl et al., 2011). Such Ca^2+^ entry is independent of SR store depletion.

In recent years, studies using genetic approaches have identified genes encoding the ER Ca^2+^ sensor, namely the stromal interaction molecule (STIM) (Liou et al., 2005; Zhang et al., 2005, also for review see Cahalan, 2009). Subsequently, the Orai family of membrane proteins was identified as forming a prototypic SOCE, the Ca^2+^ release activated Ca^2+^ channel (CRAC) (Feske et al., 2006; Vig et al., 2006; Zhang et al., 2006). CRAC channels are Ca^2+^-selective channels located in the cell membrane and fulfill the criteria for being store-operated (Feske et al., 2006; Vig et al., 2006). In response to decreased ER Ca^2+^ concentration, STIM1 translocates within the ER membrane to form discrete surface membrane-associated aggregates where it activates Orai channels (Lewis, 2007; Penna et al., 2008; Wang et al., 2010b). A recent study using high resolution nuclear magnetic resonance determined the structure of protein segments from STIM1 and Orai1 confirmed their interaction and possible role in Orai1 activation (Stathopulos et al., 2013). There is now substantial evidence that SOCE plays a key role in mediating cardiomyocyte hypertrophy, both *in vitro* and *in vivo*, and there is growing support for the contribution of SOCE to Ca^2+^ overload associated with ischemia/reperfusion injury (Collins et al., 2013). However, the expression and cellular distribution of STIM and Orai molecules have not been determined in cardiac pacemaker cells.

In this study, we examined the expression of STIM and Orai isoforms in pacemaker cells, their cellular localization under physiological conditions, and redistribution after store depletion.

## Materials and methods

### Single SAN cell isolation

Cardiac cells were harvested from male Balb/c mice (2–4 months old) under a protocol approved by the Animal Ethics Committee of the University of Sydney.

Single SAN cells were isolated using a modified protocol as described previously (Liu et al., 2011). Briefly, hearts were removed from animals and microdissection of the SAN region was performed with constant perfusion of Tyrode solution with 1.8 mmol/L Ca^2+^ using a dissecting microscope. A strip of tissue containing the SAN region, measuring ~0.5 mm × ~1 mm, was identified by anatomic landmarks and dissected out. The SAN strips were cut into 3–5 smaller strips and rinsed in a “Ca^2+^ free” solution containing (in mmol/L) 120 NaCl, 5.4 KCl, 0.5 MgCl_2_, 1.2 KH_2_PO_4_, 20 taurine, 11.1 glucose, 10 HEPES, 0.3 EGTA, 10 Na-Pyruvic acid, 5 Creatine, 5 μmol/L Blebbistatin, 2 mg/ml bovine serum albumin (BSA), pH 7.0. The rinsed SAN tissue strips were digested in the same “Ca^2+^ free” solution containing collagenase (229 u/ml, type II, Worthington Biochemical Corporation), elastase (1.9 u/ml) and protease (0.9 u/ml, type XIV) for 30–40 min at 35 ± 0.5°C and bubbled with pure oxygen. After enzyme digestion, the tissue was then washed and stored in Kruftbrühe (KB) solution which contains (in mmol/L), 30 KCl, 10 KH_2_PO_4_, 2 MgCl_2_, 70 L-glutamic acid, 10 HEPES, 20 taurine, 5 creatine, 0.3 EGTA, 10 Na-Pyruvic acid, 5 Creatine, 5 μmol/L Blebbistatin, 80 KOH, 11.1 glucose and 10 HEPES, with pH adjusted to 7.2 with KOH. Single SAN cells were released by gentle pipetting of the digested tissue strips. SAN cells were identified under light microscopy by their spindle shape and small size with centered single nuclei. Atrial myocytes were dissociated following the same isolation protocol while ventricular myocytes were dissociated by the collagenase-based coronary perfusion method as described elsewhere (Zhou et al., 2000). All chemicals were purchased from Sigma unless otherwise specified.

### Intracellular Ca^2+^ recording

Isolated single mouse SAN cells were placed on laminin-coated (20 μg/ml) 35 mm glass bottom petri dishes (MatTek Cultureware) for 20 min to attach. Fluo-4-AM (FluoroPure grade, Invitrogen. USA) was mixed with Pluronic F-127 by sonication and diluted to a final concentration of 5 μmol/L in Tyrode solution. This solution was used to load single pacemaker cells (20 min at room temperature). After superfusion with normal Tyrode solution (with 1.8 mmol/L Ca^2+^) for 20 min to de-esterify Fluo-4-AM, spontaneously beating pacemaker cells were selected for study. A LSM 510 META confocal microscope (Carl Zeiss Inc., Germany) equipped with an argon laser provided excitation at 488 nm and the fluorescence signal was collected at wavelengths of >515 nm. A heated microscope stage and a 63 × /1.4 oil objective heater (PeCon GmbH, Germany) were attached to maintain cells at 37°C throughout the experiment. Cell shortening was recorded by line scan mode and analyzed offline with ImageJ software (NIH, USA). Intracellular Ca^2+^ was recorded using either line scan mode (xt mode) or frame mode (xy mode) with time series. Results were recorded at Zeiss LSM scan speed 8 (3.07 ms/line) and 10 s intervals exist between two consecutive frames in frame mode. Data were analyzed with Zeiss LSM image examiner (version 4.2, Carl Zeiss Inc., Germany) and imageJ (NIH, USA). Global intracellular Ca^2+^ levels were translated into relative fluorescence levels, F/F0, where F and F0 represent the fluorescence intensities at a given time and at minimum resting level, respectively. Maximum change in F/F_0_ (in frame mode) was calculated as the change in the peak value after Ca^2+^ re-admission relative to initial level before Ca^2+^ removal.

### Reverse trascriptase-polymerase chain reaction (RT-PCR) analysis

Total mRNA from isolated cardiac myocytes (SAN cells, atrial and ventricular myocytes) and spleen cells were extracted with TRIzol (Invitrogen. USA) following manufacturer's protocol. Reverse transcription was carried out with 1 μg total RNA using the Superscript First-Strand Synthesis System for RT-PCR (Invitrogen), according to the manufacturer's instructions. RT-PCR was performed with Platinum Taq (Invitrogen) under the following conditions as described by Wissenbach et al. (2007): one cycle 50°C/30 min; one cycle 94°C/2 min; 40–45 cycles 94°C/15 s, 56°C/30 s, 72°C/30 s; one cycle 72°C/5 min. The following primer pairs, deduced from cDNA sequences and flanking at least one intron were used: 5′ GAT CGG CCA GAG TTA CTC C and 5′ CGA TGC ATG CGC TCG TGG (ORAI1); 5′ AA GAA GGG AGA GAC ACA CAG and 5′ ACT CGC TGA TGG AGT TGAG G (ORAI2); 5′ GCC AGT CAG CAC TCT CTG C and 5′ CCA CCA GAA CAA CTT CAG CC (ORAI3); 5′ GCC ACA GCT TGG CCT GG and 5′ GCT CCA TCA GG CTG TGG (STIM1); 5′ TGA GGA TAC CCT GCA GTG G and 5′ CAG TCT GCA GAC TCT CTA AG (STIM2); 5′ GCT CGA GAT GTC ATG AAG G and 5′ GGC TGT ACT GCT TAA CCA GG (HPRT1).

### Immunostaining and western blots

Immunostaining of single SAN cells was carried out using standard protocols as described previously (Liu et al., 2011). Briefly, isolated pacemaker cells were plated onto laminin-treated slides and allowed to settle for 30 min before being fixed with 2% paraformaldehyde (Sigma) for 5 min. A subset of cells was incubated with Ca^2+^ free Tyrode Solution with either 5 μmol/L cyclopiazonic acid (CPA) or 1 μmol/L thapsigargin (TG) for 30 min at room temperature before fixation. Fixed cells were washed three times with phosphate buffer solution (PBS) over 30 min, permeabilized by 0.1% Triton X-100 (Sigma) for 5 min, washed three times with PBS over 30 min, and blocked with 1% bovine serum albumin (BSA; Sigma) and 4% normal Goat serum (invitrogen, USA) in PBS for 1–2 h before application of primary antibody. Primary antibodies were diluted in 1% BSA and 4% normal goat serum in PBS. Cells were incubated with primary antibodies at an appropriate concentration (see Table 1) at 4°C overnight, briefly washed in PBS and then Alexa Fluo-488 goat anti-rabbit or Alexa Fluo-561 goat anti-mouse secondary antibodies (both at 1:200 dilution, Invitrogen, USA) were applied. Cells were washed three times with PBS and then mounted with Prolong Gold mounting media with DAPI (Invitrogen, USA) and cover slips were sealed with nail polish. In negative control experiments, no primary antibody was used and no labeling was detected. Confocal images were acquired with LSM 510 META confocal microscope (Carl Zeiss Inc., Germany) and analyzed with LSM image examiner.

**Table 1:** **List of antibody used in the study**.

**Antibody**	**Company (cat #)**	**Clone**	**Ab isoform**	**Dilution staining**	**Dilution western**
ORAI1	Sigma (O8264)	NA	Rabbit	1:100	
ORAI1	ProSci (PM-5207)	NA	Mouse	1:500	1:1000
ORAI3	ProSci (4117)	Polyclone	Rabbit	1:200	1:1000
STIM1	BD transduction	44/stim1	Mouse IgG2a	1:100	1:1000
STIM2	ProSci (4123)	Polyclone	Rabbit	1:400	1:1000
HCN4	Abcam (ab32675)	SHG 1E5	Rat	1:800	
Caveolin-3	BD transduction	26/cav-3	Mouse IgG1	1:500	
SERCA2	Abcam (ab2861)	2A7-A1	Mouse IgG2a	1:500	
RyR2	ABR (MA3-916)	C3-33	Mouse IgG1	1:200	
NCX1	Swant (R3F1)	R3F1	Mouse IgG1	1:200	

For Western blot, strips of SAN (restricted by Crista terminalis, atrial septum, superior and inferior vena cava), atrium (from left atrium wall) and ventricle (left ventricle free wall) were freshly dissected and snap frozen in liquid nitrogen. Spleen tissue was collected in the same way and served as positive control protein. Total protein extracts were prepared with Mammalian Cell Lysis Kit (Sigma, Cat no: MCL1) containing RIPA buffer and protease inhibitor cocktail. Tissue samples were homogenized on ice using a Polytron homogenizer (PT900 CL) and cleared by centrifugation at 12,000 g for 10 min. Protein extracts were separated on 8% SDS-PAGE gel and transferred to a nitrocellulose membrane (Whatman). The membranes were immunoblotted with the appropriate antibodies (Table 1) following standard procedures published elsewhere (Nishiyama et al., 2009). Immunoblots were probed with antibodies against STIM1, STIM2, Orai1, and Orai3 but not Orai2 due to lack of Orai2 mRNA expression in cardiac cells.

### Drugs

Thapsigargin (TG, 1 μmol/L) and Cyclopiazonic acid (CPA, 5 μmol/L) which are SR Ca^2+^-ATPase (SERCA) inhibitors were used to deplete store content. The imidazole compound SKF-96365 (1-[2-(4-methoxyphenyl)-2-[3-(4-methoxyphenyl) propoxy]ethyl-1H-imidazole hydrochloride) has been shown to target the STIM1-Orai1 pathway and inhibit SOCE in cell lines (Liou et al., 2005; Huang et al., 2006). BTP-2, a bistrifluoromethyl-pyrazole derivative, is a potent and fast-acting SOCE blocker in a number of immortal cell lines and immune cells (He et al., 2005; Yonetoku et al., 2008). SKF-96365 and BTP-2 (both at 10 μmol/L) were used as SOCE blockers in this study. All drugs were dissolved in DMSO as stock solution stored at −20°C and diluted in Tyrode solution at working concentration before applying to cells.

### Statistical analysis

Data were presented as means ± SEM. The statistical significance of effects was evaluated by Student's *t*-test or ANOVA and a value of *P* < 0.05 was considered statistically significant.

## Results

### SOCE activities in single isolated pacemaker cells

**Figure 1:**
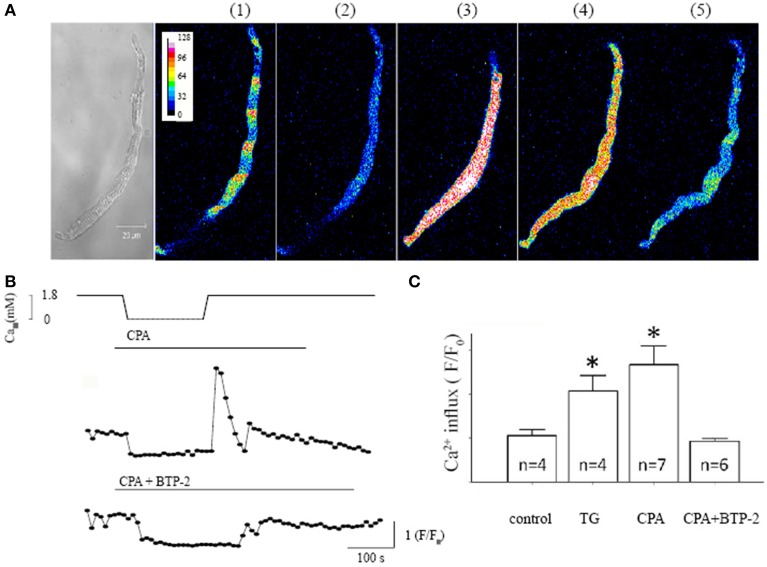
**Characteristic store operated Ca^2+^ entry (SOCE) induced in isolated pacemaker cells. (A)** The left panel shows a transmitted light image of a spontaneously firing pacemaker cell loaded with Ca^2+^ indicator Fluo-4 AM. The regular spontaneous Ca^2+^ transients were detected as regular fluctuations in fluorescence intensity during a single xy frame recorded over a period of 1.5 s, (during which there were ~5 beats under control condition as shown next image (1). There are five selected fluorescent images representing intracellular Ca^2+^ changes under 5 different experimental conditions in the same cell; (1) control, (2) after store depletion with 0 mmol/L Ca^2+^ and the SERCA inhibitor cyclopiazonic acid (CPA, 5 μmol/L), (3 and 4) upon Ca^2+^ re-admission, and (5) wash off respectively. **(B)** The time course of Ca^2+^ fluorescent intensity changes deduced from 60 Ca^2+^ images consecutively collected at 10 s intervals. The top panel shows the course of SOCE activation protocol, i.e., timing of extracellular [Ca^2+^] withdrawal and readmission and time of application of CPA. The bottom trace shows the intracellular Ca^2+^ changes in the presence of SOCE blocker BTP-2 during the course of SOCE activation. **(C)** Summary data (mean ± SEM) represents the maximum amplitude of [Ca^2+^]_i_ rise upon Ca^2+^ re-admission, relative to the initial [Ca^2+^]_i_ level before Ca^2+^ removal. Control experiments were done with Ca^2+^ removal and re-admission only. N equals number of cells for each group, ^*^P < 0.05.

To investigate whether SOCE exists in isolated single pacemaker cells, we studied the changes in [Ca^2+^]_i_ in response to an intervention protocol which involves removing extracellular Ca^2+^ in addition to the application of SR Ca^2+^-ATPase (SERCA) inhibitor. We previously showed that this protocol caused activation of SOCE in intact mouse SAN tissue (Ju et al., 2007). Figure 1A shows serial confocal Ca^2+^ images collected from an isolated single isolated pacemaker cell undergoing the SOCE activation protocol. Under control conditions (1.8 mM [Ca^2+^]_o_), the cell exhibited spontaneous firing and regular Ca^2+^ transients Figure 1A(1) (xy plot, duration 1.5 s). The cell stopped firing when the SERCA inhibitor, cyclopiazonic acid (CPA, 5 μmol/L) was applied along with extracellular Ca^2+^ withdrawal from the solution for 5 min, which was associated with a significantly reduced [Ca^2+^]_i_ [Figure 1A(2)]. A marked global rise in [Ca^2+^]_i_ upon Ca^2+^ re-admission is demonstrated in Figure 1A(3) associated with visible cell shortening (hypercontraction). [Ca^2+^]_i_ rapid declined after an initial transient overshoot [Figure 1A(4)]. Spontaneous Ca^2+^ transients associated with pacemaker firing reappeared as [Ca^2+^]_i_ gradually returned to control level after wash off of CPA [Figure 1A(5)]. Figure 1B demonstrated the time course of Ca^2+^ fluorescence intensity changes deduced from 60 consecutively collected Ca^2+^ images in response to the SOCE activation protocol. The top panel showed the timing of extracellular [Ca^2+^] withdrawal and readmission. A large Ca^2+^ influx reached its maximum within 30 s after Ca^2+^ re-admission and lasted for 1–2 min (Figure 1B top trace). In contrast, only a small Ca^2+^ influx was seen in the presence of the selective SOCE blocker BTP-2 (Yonetoku et al., 2008; Singh et al., 2010) (Figure 1B bottom trace). We found that SKF-96365 has similar effect to BTP-2 in single pacemaker cells and the results were similar to that previously reported in intact SAN preparations (Ju et al., 2007). Both SOCE blockers also significantly reduced CPA or TG -induced SOCE in single pacemaker cells. In addition, Ca^2+^ influx was not seen upon reintroducing extracellular Ca^2+^ after removal of extracellular Ca^2+^ without using SERCA inhibitors. Figure 1C compares the amplitude of the [Ca^2+^]_i_ rise upon Ca^2+^ re-admission relative to initial levels for each treatment. These results confirm there is significant Ca^2+^ influx in isolated single mouse pacemaker cells in response to a SOCE activation protocol that presumably causes store depletion.

**Figure 2:**
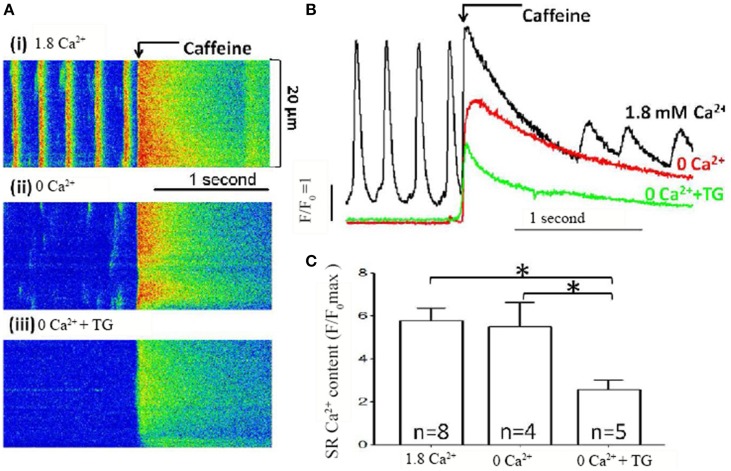
**SR Ca^2+^ store content measured in pacemaker cells. (A)** Representative line scan recording of [Ca^2+^]_i_ and caffeine induced Ca^2+^ transients, as the measurement of SR Ca^2+^ store content, in SAN cells; (i) under control conditions with 1.8 mmol/L Ca^2+^, (ii) with removal of external Ca^2+^ only and (iii) with removal of Ca^2+^ in the presence of TG. **(B)** Overlapping representative line plot traces of Ca^2+^ images shown in **(A)**. The associated line colors represented [Ca^2+^]_i_ under control conditions (black), removal of external Ca^2+^ only (red) and removal of Ca^2+^ in the presence of TG (green), respectively. **(C)** Summary data (mean ± SEM) of store content indicated by maximal rise of F/F_0_ measured by caffeine induced Ca^2+^ release. N equals number of cells for each group, ^*^P < 0.05.

To confirm that the large [Ca^2+^]_i_ rise was indeed associated with a store Ca^2+^ depletion, the store Ca^2+^ content was assessed using rapid caffeine application (Figure 2). Rapid application of caffeine causes a large and rapid rise in [Ca^2+^]_i_ that has been widely used to measure SR Ca^2+^ content (Bers, 1987). Caffeine-induced Ca^2+^ transients were compared under control conditions [Figure 2A(i)], after 5 min. of extracellular Ca^2+^ removal [Figure 2A(ii)], and after Ca^2+^ removal combined with SERCA inhibition by TG [Figure 2A(iii)]. Three superimposed spatially-averaged traces of caffeine-induced Ca^2+^ transients are shown in Figure 2B. Note that regular spontaneous Ca^2+^ transients were seen only in control conditions before the application of caffeine (black line, Figure 2B), but not in extracellular Ca^2+^ free (red line) and extracellular Ca^2+^ free plus thapsigargin (green line). SR Ca^2+^ store content indicated by caffeine induced Ca^2+^ transients (quantified by maximum ΔF/F_0_) showed a small but non-significant reduction after extracellular Ca^2+^ removal (Figure 2C, *n* = 4 cells from 3 mice, *P* = 0.81) but exhibited a large and significant fall when extracellular Ca^2+^ removal was combined with SERCA inhibition (Figure 2C, *n* = 5 cells from 3 mice, *P* < 0.05). These data confirm that the combination of extracellular Ca^2+^ removal and application of a SERCA blocker cause store depletion. The large Ca^2+^ influx is dependent on SR Ca^2+^ store content and is store-operated Ca^2+^ entry.

### SOCE blockers reduce store content

The important physiological function of SOCE is to refill the SR Ca^2+^ store (Seth et al., 2012). Thus, it would be expected that SOCE blockers would cause decreased SR Ca store content.

**Figure 3:**
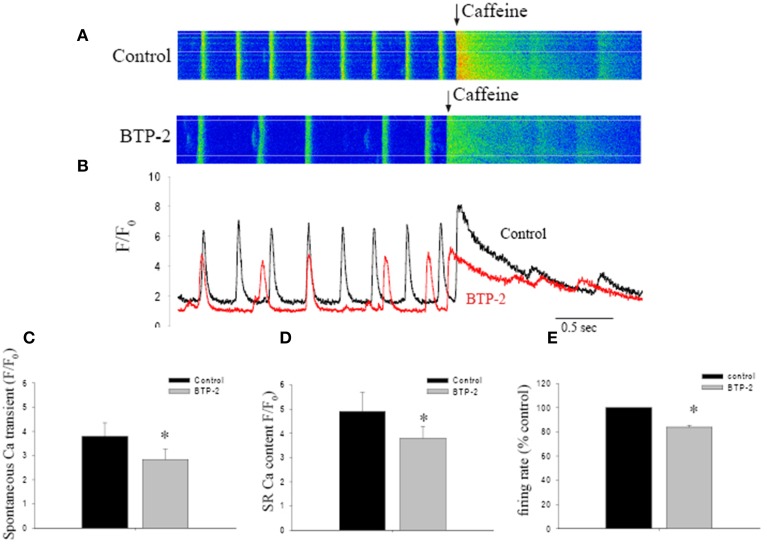
**The effect of BTP-2, a SOCE inhibitor on the spontaneous firing rate, Ca^2+^ transient amplitude and SR Ca^2+^ content in pacemaker cells. (A)** Confocal Ca^2+^ images show the changes in firing rate and [Ca^2+^]_i_ before and after BTP-2 application. **(B)** The line plots deduced from images in **(A)** that compare spontaneous Ca^2+^ transients and SR store content (caffeine induced Ca^2+^ transients) before and after BTP-2 application. **(C)** Summary data (mean ± SEM) represents average change of ΔF/F_0max_ of spontaneous Ca^2+^ transients, SR Ca^2+^ content and pacemaker firing rate in control, BTP-2, respectively. ^*^P < 0.05.

To test this idea, we examined the effect of a SOCE blocker on SR Ca^2+^ store content, estimated by caffeine-induced Ca^2+^ release (Bers, 1987). Figure 3A shows Ca^2+^ transients recorded from a spontaneously beating pacemaker cell. Rapid application of caffeine induced a large and rapid rise in [Ca^2+^]_I_ under control conditions. BTP-2 slowed the spontaneous firing rate and reduced the amplitude of both the spontaneous Ca^2+^ transient and the caffeine-induced transient. Both these observations are clearly shown in Figure 3B which shows superimposed, spatially-averaged records. On average BTP-2 reduced the amplitude of the spontaneous Ca^2+^ transient by 24% (*n* = 6, *P* = 0.018, Figure 3C). BTP-2 also reduced the amplitude of the caffeine-induced Ca^2+^ signal—which is indicative of SR Ca^2+^ store content—by 22% (*n* = 6 cells from 4 mice, *P* = 0.026) (Figure 3D). These data demonstrate SOCE inhibition reduced store content and firing rate, which suggests that SOCE may participate in maintaining store content and pacemaker function. To estimate how much SOCE might be involved in normal pacemaker activity, we investigated the effect of SOCE blockers on pacemaker firing rate. When applied to normally firing pacemaker cells, BTP-2 reduced the firing rate by 16.0 ± 1.4% (*n* = 6 cells in each group, *P* < 0.01 as shown in Figure 3E). SKF-96365 also reduced pacemaker firing rate by 12.3 ± 1.8%. The negative chronotropic effects caused by inhibition of SOCE may indicate that SOCE might be involved in the regulation of pacemaker activity.

### Expression of STIM/Orai in pacemaker cells

**Figure 4:**
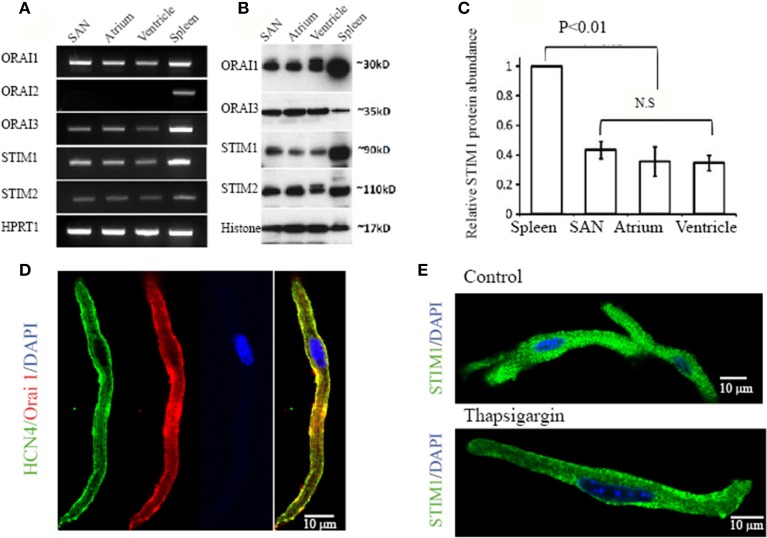
**Expression of STIM and Orai molecules in mouse cardiac myocytes. (A)** Total RNA was isolated from pacemaker cells (SAN), atrial myocytes (Atrium), ventricular myocytes (Ventricle) and spleen. Transcripts of Stim1, Stim2, Orai1, Orai2, and Orai3 in cardiac myocytes were compared with the spleen as positive control. The housekeeping gene Hypoxanthine–Guanine Phosphoribosyltransferase (HPRT1) was used as an internal control. **(B)** Protein levels of STIM1, STIM2, Orai1, and Orai3 were examined in total protein extracts from SAN region (SAN), atrium (Atrium), ventricle (Ventricle) and spleen and determined by Western Blot analysis. The nuclear protein histone was used as loading control. **(C)** Bar graph showing quantitative western blot analysis of STIM1 protein levels in spleen and heart with heart samples normalized to spleen as 1 (*n* = 3 gels, *P* < 0.05, ANOVA). **(D)** Confocal images of an isolated pacemaker cell shows expression of HCN4 (green in color), a positive marker for pacemaker cells. The cell also positively stained with anti-Orai1 antibody (red in color). The nucleus is stained blue with DAPI. A merged image on the left shows the co-localization of HCN4 and Orai1 along the surface membrane (color in yellow). **(E)** Representative confocal images of an isolated pacemaker cell labeled with STIM1 (green in color) and DAPI (blue in color). When perfused with normal Ca^2+^ containing solution, positive STIM1 staining was distributed both intracellularly and in the cell periphery as showed in the top panel. When perfused with Ca^2+^-free solution containing thapsigargin, STIM1 peripheral localization was enhanced, as shown in the bottom panel.

Although we found SOCE activity in pacemaker cells as described above, the genes encoding SOCE in pacemaker cells remain to be identified. In recent years, Orai proteins have emerged as new molecular candidates for the channels that underlie the store depletion activated Ca^2+^ current, I_CRAC_ (Huang et al., 2006; Zhou et al., 2010). Before the discovery of Orai genes, the TRPC channels were regarded as the most likely candidates for SOCE. We previously found that pacemaker cells express all TRPC isoforms, except TRPC5 (Ju et al., 2007). However, it is still debatable whether the activation of TRPC channels is dependent on Ca^2+^ depletion from SR Ca^2+^ store (SOCE) or is dependent on G-protein coupled receptor activation (ROCE) (Hofmann et al., 1999). In addition, stromal interacting molecular 1 (STIM1) has been identified as the ER sensor. It is known that activation of SOCE requires STIM1 migration and interaction with other molecular components of SOCE (Lewis, 2007). Given the importance of STIM1 and Orai, we wanted to establish their expression in pacemaker tissue. RT-PCR was performed with mRNA extracted from isolated SAN, atrial and ventricular myocytes, respectively. Spleen tissue was used as a positive control sample, as there is abundant expression of these genes in immune cells. The housekeeping gene Hypoxanthine–Guanine Phosphoribosyltransferase (HPRT) was used as an internal control. Figure 4A shows gel images of amplified PCR products. SAN and other cardiac myocytes expressed mRNA transcripts of STIM1, STIM2, Orai1, and Orai3, but not Orai2. In contrast, spleen tissue expressed all STIM and Orai isoforms as reported by others (Wissenbach et al., 2007).

Expression of STIM and Orai proteins in SAN and other cardiac tissue were also examined by immunoblot with specific antibodies against each isoform. Figure 4B shows bands of STIM1, STIM2, Orai1, and Orai3 in cardiac tissues at their predicted molecular weight. The antibodies recognize proteins with appropriate molecular weight and confirm the expression of STIM1, STIM2, Orai1, and Orai3 in the heart tissues, including SAN, atrial and ventricular tissue. Figure 4C shows quantitative western blot analysis of STIM1 protein levels in spleen and heart with heart samples normalized to spleen.

Among the different isoforms of STIM and Orai, the STIM1 and Orai1 have been considered to be the most relevant components of SOCE (Lewis, 2007; Stathopulos et al., 2013). To explore the possibility that STIM1 and Orai1 in pacemaker cells are functioning in SOCE, we investigated the localization of STIM1 and Orai1 proteins in isolated SAN cells using immunohistochemistry. It is known that pacemaker cells express HCN4 (hyperpolarization-activated, cyclic nucleotide-gate cation channels, type 4) (Marionneau et al., 2005; Liu et al., 2007), a channel carrying I_f_current in these cells (Herrmann et al., 2007). Figure 4D shows that a HCN4 positive cell (green in color) isolated from mouse SAN tissue also demonstrates positive labeling of Orai1 (red in color). The distribution of Orai1 labeling is enhanced in the surface membrane where HCN4 is also located (yellow color showed in merged images). This result suggests that Orai1 could form functional channels in pacemaker cells. We found that positive HCN4 labeled cells can be also positively labeled with anti-STIM1 antibody (data not shown). STIM1 is an SR/ER Ca^2+^ sensor protein located predominantly within the ER/SR, but also to a limited extent in the plasma membrane (Liou et al., 2005; Wu et al., 2006). When perfused with normal Ca^2+^ containing solution, the distribution of STIM1 staining was mainly intracellular as showed in two isolated SAN pacemaker cells (Figure 4E top panel). It has been reported that STIM1 can be translocated toward the plasma membrane upon SR Ca^2+^ store depletion in transfected RBL cells (Liou et al., 2007). After treating pacemaker cells with thapsigargin, we found that the STIM1 staining was redistributed and became predominately located at the cell membrane as shown in Figure 4E, bottom panel.

### Peripheral redistribution of STIM1 upon depletion of SR Ca^2+^ store

**Figure 5:**
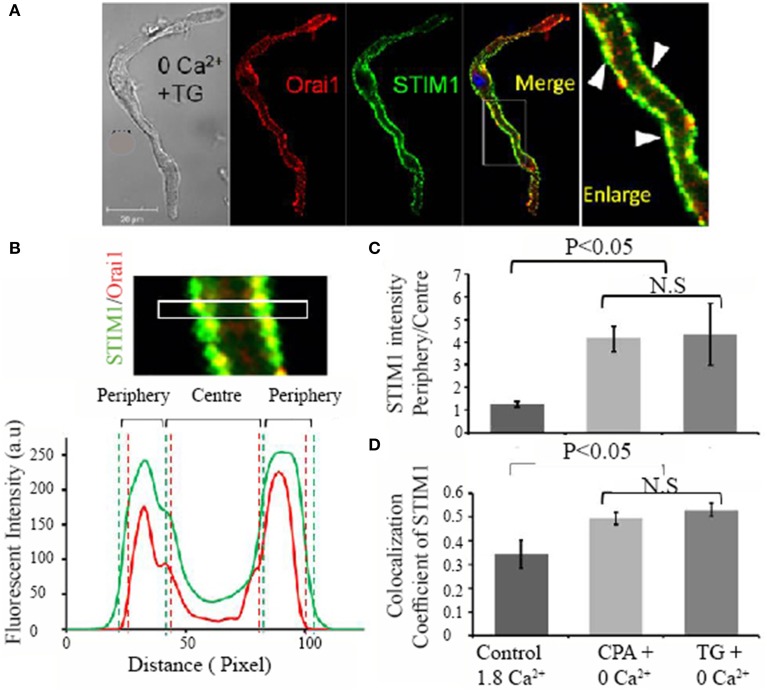
**Evidence of migration of STIM1 proteins to the cell membrane region after store depletion**. Ca^2+^ store was depleted by treating cells with Ca^2+^ free solution plus 10 μmol/L thapsigargin. **(A)** The colocalization of STIM1 and Orai1 is demonstrated by membrane staining of STIM1 in the merged image (yellow in color) and is also indicated by white triangle arrows in the enlarged image (far right panel). To determine the region of a cell, the confocal image of a cell was equally divided into peripheral quarters and center quarters across cell width, and fluorescent intensity in each quarter was plotted against distance as demonstrated in **(B)**. **(C)** Summary data of the fluorescent intensity ratio of peripheral quarters to center quarters of STIM1 under three conditions; control, CPA + 0 Ca^2+^, thapsigargin (TG) + 0 Ca^2+^ (*n* = 9 cells in each group, *P* < 0.05, ANOVA). **(D)** Summary data (mean ± SEM) of the colocalization coefficiency, a ratio of STIM1 and Orai1 colocalized pixels (Colocal) over total STIM1 pixels under three conditions. (*n* = 9 cells in each group, *P* < 0.05, ANOVA).

Store-dependent translocation of STIM1 appears to be a prerequisite for the physical interaction with Orai1, which is followed by Orai1 activation and Ca^2+^ influx (Liou et al., 2007). To further investigate whether such STIM1 translocation can form a functional interaction with Orai1 in pacemaker cells, we examined the localization of Orai1 and STIM1 under control and store depleted conditions. Figure 5A shows a pacemaker cell that was immersed in Ca^2+^ free solution containing thapsigargin before labeling with Orai1 (red) and STIM1 (green). Orai1 labeling appears closer to the cell membrane, while STIM1 labeling also appears along the surface membrane where Orai1 is located. The colocalization of two proteins is demonstrated by the yellow staining as showed in an enlarged panel (arrows) [Figure 5A(ii)]. We examined two separate groups of pacemaker cells, the control group cells that were kept in normal Tyrode solution (Ca^2+^ 1.8 mmol/L) whereas the store depleted cells were kept in Ca^2+^ free solution containing the SERCA blockers CPA or TG for 30 min. The translocation after store depletion was quantified by dividing the diameter of cells into 4 equal regions (Figure 5B) and the ratio of two outer quartiles (including cell membrane) to two inner quartiles (cell interior) was calculated (Figure 5B). For Orai1, this ratio is around 2–3, indicating substantial concentration in the cell membrane, and does not change with store depletion (data not shown). Conversely for STIM1, the ratio in control cells is 1.25 ± 0.12 (*n* = 9 cells in 1.8 mM Ca^2+^), indicating very little concentration in the cell membrane, but changed significantly to 4.16 ± 0.56 (*n* = 9 in the presence of CPA) or 4.35 ± 1.38 (*n* = 9 in the presence of TG) (*P* < 0.05) when the store was depleted (Figure 5C). This data shows increased peripheral localization of STIM1 and increased colocalization of STIM1 and Orai1 upon store depletion. Under the same conditions, images of these antibody-stained cells were analyzed for colocalization, utilizing the colocalization coefficient (the ratio of colocalized STIM1 pixels to total STIM1 pixels). Colocalization coefficients for both STIM1 increased significantly after store depletion (Figure 5D, *n* ≥ 9 cells per group, *P* < 0.05). Similar results were obtained for the colocalization of Orai1 with STIM1 (not shown). This increased colocalization resulting from the peripheral redistribution of STIM1 is expected to increase the opportunity for physical interaction between STIM1 and Orai1, resulting in activation of calcium influx through Orai1.

## Discussion

### SOCE and pacemaker function

In the present study, we examined both the functional consequences of SOCE and molecular components of SOCE in isolated pacemaker cells. The study strengthens our earlier identification of SOCE in intact mouse SAN (Ju et al., 2007).

Given that pacemaker cells generate robust spontaneous firing by a combination of voltage-dependent channels and Ca^2+^ cycling, one might question what functional role SOCE can play in pacemaker cells. Application of the blockers to the normally firing pacemaker cells caused only modest reductions of the firing rate, though the fact that they were accompanied by a reduction in store level strengthens the case for the effect arising from blocking SOCE. Reduced SR store Ca^2+^ content would influence both diastolic and systolic SR Ca^2+^ release and hence influence pacemaker activity (Bassani et al., 1995; Vinogradova et al., 2004, 2006). However, we could not eliminate the possible effect of these blockers on other pacemaker currents such as voltage-gated Ca^2+^ current or potassium currents, which might prevent us from accurately estimating the contribution of SOCE to pacemaker electrical activity.

From an evolutionary perspective, survival of an animal depends critically on the activity of several small groups of pacemaker cells. No doubt this is the reason why pacemaker cells contain several independent pacing mechanisms so that the loss of one through ingested toxin, injury or genetic mutation is not necessarily lethal. The experimental data presented here demonstrated a small change (~15% decrease) in firing rate when SOCE was blocked, and thus supports a possible role of SOCE in regulating the firing rate under physiological conditions. Using computer simulation, we recently found that an additional inward SOCE current with a long time constant of activation (e.g., 800 ms) could lead to a small increase (~11%) in firing rate. With such a long activation time constant, SOCE current effectively becomes a background current whose level changes little over the cardiac cycle and whose magnitude depends on the mean level of Ca^2+^ in the SR over the cardiac cycle, providing a calcium influx regulated by mean store size (Allen et al., 2012). Background currents of this sort have been previously recognized in rabbit pacemaker cells (Hagiwara et al., 1992). In this context, it would be of interest to determine whether the background current in pacemaker cells is dependent on SR Ca^2+^ store depletion.

### Distribution and localization of STIM and Orai in pacemaker cells

In the present study we demonstrate that SOCE activity is present in single pacemaker cells. We also demonstrate that several new SOCE proteins, including STIM1, STIM2, Orai1, and Orai3, but not Orai2, are expressed in pacemaker cells of mouse SAN tissue. Using immunohistochemistry, we show that while Orai1 proteins are predominantly located in the sarcolemma, STIM1 proteins under control conditions are distributed across the cell. It has been reported that STIM activation induces conformational changes of Orai proteins and subsequent STIM-Orai colocalization, which forms the active store-operated calcium channel (Lewis, 2007; Stathopulos et al., 2013). We also found that there was a certain proportion of STIM1 and Orai1 clustered and co-localized at the periphery of pacemaker cells in response to the store depletion. These results suggest a possible interaction between STIM1 and Orai1 and how they might play a functional role related to SOCE. However, to further quantify the involvement of STIM1 and Orai1 in SOCE activities in native pacemaker cells would require the use of tissue specific/conditional knock out related genes and proteins and then record associated changes of the amplitude of Ca^2+^ release activated current (I_CRAC_).

While the molecular mechanism of STIM1/Orai1 activity and their functional importance have been studied in great detail, the functional relevance of other isoforms of STIM and Orai still remain speculative (Hoth and Niemeyer, 2013). STIM2 and STIM1 share 60% homology in their amino acid sequence. Although we also found the presence of STIM2, the role of STIM2 in pacemaker cells might be less significant as we did not observe the translocation of STIM2 in response to store depletion (data not shown).

We also found that in addition to Orai1, Orai3 was also expressed in cardiac myocytes, including pacemaker cells, a result consistent with a recent finding in rat ventricular myocardium (Wolkowicz et al., 2011). In addition to forming functional SOCE channels, STIM1 and Orai1 have been shown to interact with many other Ca^2+^ handling proteins, including TRPC (Liao et al., 2008, 2009), the L-type Ca channel (Wang et al., 2010a), the sodium-calcium exchanger (Liu et al., 2010), the plasma membrane Ca^2+^ ATPase (Ritchie et al., 2012), and the sarcoplasmic reticulum Ca^2+^ ATPase (Lopez et al., 2008). Given that all these potential pathways could contribute to Ca^2+^ handling and therefore pacemaker function, these offer multiple possible directions for future research.

In our earlier work we identified that TRPC1, 3, 4, and 6 are all expressed in pacemaker cells (Ju et al., 2007). The current study demonstrates the presence of two of the best characterized SOCE components, STIM1 and Orai1, in pacemaker cells and in addition demonstrates translocation of STIM1 to the sites of Orai1 on store depletion, as characterized in many other cell types. It has been suggested that Orai and TRPC protein form complexes that participate in Ca^2+^ entry with or without activation of store depletion (Liao et al., 2009). Since these SOCE related proteins are all expressed in the pacemaker cells, it is possible that SOCE or related Ca^2+^ signaling pathways such as receptor operated Ca^2+^ entry (ROCE) (Hofmann et al., 1999) might contribute to pacemaker activity under physiological or pathophysiological conditions.

## Limitations of the current study

A patch clamp study is needed to further establish whether SOCE is accompanied by an inward current (Potier et al., 2009) which directly contributes to diastolic pacemaker potential.

### Conflict of interest statement

The authors declare that the research was conducted in the absence of any commercial or financial relationships that could be construed as a potential conflict of interest.
